# Obesity-resistance of UCP1-deficient mice associates with sustained FGF21 sensitivity in inguinal adipose tissue

**DOI:** 10.3389/fendo.2022.909621

**Published:** 2022-08-11

**Authors:** Marlou Klein Hazebroek, Susanne Keipert

**Affiliations:** Department of Molecular Biosciences, The Wenner-Gren Institute, Stockholm University, Stockholm, Sweden

**Keywords:** FGF21 resistance, beige fat, diet induced obesity, beta klotho, browning, FGF21 sensitivity

## Abstract

Metabolic diseases represent the major health burden of our modern society. With the need of novel therapeutic approaches, fibroblast growth factor 21 (FGF21) is a promising target, based on metabolic improvements upon FGF21 administration in mice and humans. Endogenous FGF21 serum levels, however, are increased during obesity-related diseases, suggesting the development of FGF21 resistance during obesity and thereby lowering FGF21 efficacy. In uncoupling protein 1 knockout (UCP1 KO) mice, however, elevated endogenous FGF21 levels mediate resistance against diet-induced obesity. Here, we show that after long-term high fat diet feeding (HFD), circulating FGF21 levels become similarly high in obese wildtype and obesity-resistant UCP1 KO mice, suggesting improved FGF21 sensitivity in UCP1 KO mice. To test this hypothesis, we injected FGF21 after long-term HFD and assessed the metabolic and molecular effects. The UCP1 KO mice lost weight directly upon FGF21 administration, whereas body weights of WT mice resisted weight loss in the initial phase of the treatment. The FGF21 treatment induced expression of liver *Pck1*, a typical FGF21-responsive gene, in both genotypes. In iWAT, FGF21-responsive genes were selectively induced in UCP1 KO mice, strongly associating FGF21-sensitivity in iWAT with healthy body weights. Thus, these data support the concept that FGF21-sensitivity in adipose tissue is key for metabolic improvements during obesogenic diets.

## Introduction

Fibroblast growth factor 21 (FGF21) is considered a promising therapeutic agent for obesity and related diseases (1, 2). FGF21 is a hormone with pleiotropic effects on energy homeostasis, secreted by and acting on multiple organs (3). FGF21 binds specifically to the FGF receptors (FGFR) but requires beta Klotho (KLB) as an obligate co-receptor (4, 5). While FGFRs are ubiquitously expressed, KLB expression is limited to metabolic tissues such as liver, white and brown adipose tissues (WAT and BAT), pancreas and distinct brain regions, conferring specificity of FGF21 signaling (3). FGF21 signals during cellular and environmental stress responses, such as starvation, to promote gluconeogenesis and ketogenesis, thereby facilitating the adaptation to stress (6, 7). The administration of exogenous FGF21 in mice and primates improves obesity-related insulin resistance, glucose and lipid homeostasis, further suggesting a major role in the regulation of systemic metabolism (8–11).

First generation FGF21 analogues have already reached the clinical test phase (12, 13). While the effects on body weight loss and glucose homeostasis are rather small, significant improvements of dyslipidemia and fatty liver are seen in patients with obesity and type 2 diabetes (T2D) (9, 13–15). Thus, preclinical and clinical evidence corroborate that FGF21 is a promising candidate to combat obesity related diseases. Since FGF21 corrects multiple metabolic disorders by improving lipid metabolism and body weight (10, 14–16), one would expect rather low blood levels of FGF21 during metabolic disease progression. Surprisingly, however, Zhang and colleagues found in 2008 that serum FGF21 was significantly increased in obese patients and associated with increased risk of the metabolic syndrome, suggesting FGF21 as potential biomarker for metabolic diseases (17). Importantly, these observations started discussions whether obesity may cause FGF21 resistance. Numerous subsequent studies confirmed the increase of endogenously circulating FGF21 levels during obesity and metabolic disease in mice and humans (17–20). Recently it was reported that FGF21 is postprandially regulated by insulin rather than glucose, an effect that is attenuated in patients with T2D (21). Whether systemic FGF21 resistance exists or even promotes insulin resistance, is controversially discussed (2, 7, 19, 22, 23).

Several lines of evidence implicate adipose tissue as the important regulator of FGF21 action, mediating its pleiotropic metabolic effects (8, 24–26). In white adipocytes, FGF21 promotes insulin-independent glucose uptake, regulates lipolysis, and increases mitochondrial biogenesis (8, 27). Interestingly, the expression of its co-regulator KLB is repressed in adipose tissues of dietary-obese mice (19, 25, 28, 29) and obese humans (28). Mice with the adipocyte-specific deletion of FGFR1 or KLB are resistant to the therapeutic benefits of FGF21 administration (30, 31). Furthermore, the overexpression of KLB in adipose tissue is sufficient to enhance FGF21 responses and to combat dietary obesity in mice (29). In contrast, others observed that the maintenance of KLB in adipose tissue does not increase FGF21 sensitivity *in vivo* (25). Therefore, it remains unclear how FGF21 sensitivity in adipose tissue contributes to metabolic improvements during FGF21 therapy. Still, the majority of the recent studies support a concept that FGF21 action and sensitivity are required in adipose tissue for metabolic improvements during obesity and insulin resistance.

In contrast to elevated levels of endogenous FGF21 serum levels during obesity and obesity-related diseases, high endogenous FGF21 levels in mouse models with genetically modified mitochondrial function improve metabolism and mediate resistance to diet-induced obesity (7). Endogenous FGF21 fully mediates obesity resistance in mice with genetic inactivation of the adipose-specific mitochondrial uncoupling protein 1 (UCP1), when fed high fat diets (HFD) at room temperature (representing mild cold conditions) (32). The phenomenon of increased FGF21 levels and resistance to diet induced obesity in UCP1 KO mice is only seen at mild cold, not thermoneutral conditions (33, 34). To date, the underlying mechanisms how FGF21 counteracts obesity are not understood but associate with the browning of WAT.

In this study, we show that long term HFD feeding at room temperature elevates endogenous serum FGF21 levels in wildtype (WT) mice to comparable levels of their UCP1 KO littermates. The administration of exogenous FGF21 acutely reduces body weights only of UCP1 KO mice that display improved FGF21 sensitivity of iWAT, which may be the driving force for maintaining the healthy metabolic phenotype. On the contrary, the wildtype data further support adipose tissue-specific resistance to FGF21 action upon HFD feeding. The potential impact of adipose tissue-selective FGF21-resistance may have important ramifications for the therapeutic effects of FGF21 analogous.

## Methods

### Animals

The experiments were performed in homozygous male WT, UCP1 KO, FGF21 KO and UCP1/FGF21 double KO (dKO) mice (genetic background C57BL/6J). To reduce confounding developmental adaptation to thermal stress we bred, raised and maintained all mice at thermoneutrality. Mice were housed in groups with ad libitum access to food and water, and a 12:12-h dark–light cycle. At the age of ~10–12 weeks, mice were changed to a 58% high fat diet (Research diets, D12331) and housing temperature was changed from 30°C to 23°C ± 1°C. Body composition was analyzed using a magnetic resonance whole-body composition analyzer (Echo-MRI). After 12 weeks of dietary intervention, mice were sacrificed 3–4 h after lights went on, and serum and tissue samples were collected. In a second cohort (identical conditions to cohort 1), of 11 weeks HFD fed male WT and UCP1 KO mice, hFGF21 (1mg/kgBW/day) was injected intraperitoneally once daily for consecutive 10 days. The animal welfare authorities of the local animal ethics committee of the state of Bavaria (Regierung Oberbayern) and North Stockholm approved animal maintenance and all experimental procedures in accordance with European guidelines.

### Gene expression analysis

RNA was extracted using Qiazol according to the manufacturer’s instructions (Qiagen). Synthesis of cDNA and DNase treatment were performed from 1 μg of total RNA using QuantiTect Reverse Transcription Kit (Qiagen). For real time qPCR performed with SYBRgreen (Applied Biosystems or Biorad), oligonucleotide primer sequences for the following genes were used: *Hprt*: for-CAGTCCCAGCGTCGTGATTA, rev-AGCAAGTCTTTCAGTCCTGTC; *B2m*: for-CCCCACTGAGACTGATACATACGC, rev-AGAAACTGGATTTGTAATTAAGCAGGTTC; *Fgfr1*: for-TGTTTGACCGGATCTACACACA, rev-CTCCCACAAGAGCACTCCAA; *Fgf21*: for-CTGCTGGGGGTCTACCAAG, rev-CTGCGCCTCCACTGTTCC; *Klb*: for-AACCAAACACGC GGATTTC, rev-GATGAAGAATTTCCTAAACCAGGTT; *Ucp1*: for-GGCCTCTACGACTCAGTCCA, rev- TAAGCCGGCTGAGATCTTGT; *Dio2*: for-AATTATGCCTCGGAGAAGA, rev-GGCAGTTGCCTAGTGAAAG; *Cidea*: for-AATGGACACCGGGTAGTAAGT, rev-CAGCCTGTATAGGTCGAAGGT; *Pck1*: for-TGCATAACGGTCTGGACTTC, rev-CAGCAACTGCCCGTACTCC. Gene expression was calculated as ddCT using *Hprt* or *B2m* for normalization. The data are shown as values relative to the WT group.

### Serum analysis

FGF21 serum levels were measured according to the manufacturer’s instructions (Mouse/Rat FGF21 ELISA Kit - R&D Systems).

### Statistic

Statistical analyses were performed using Stat Graph Prism 9 (GraphPad Software, San Diego, CA USA). 1-way or 2-way ANOVA and Sidak’s multiple comparisons test were used to determine differences between the genotypes. Statistical significance was assumed at p < 0.05. Statistical significance of WT to other genotypes are denoted by *p < 0.05, **p < 0.01, ***p < 0.001. Statistical differences between the treatment within one genotype are indicated #.

## Results

### Sustained FGF21 receptor complex expression in UCP1 KO mice despite the lack of genotype difference in circulating FGF21 after long term HFD

**Figure 1:**
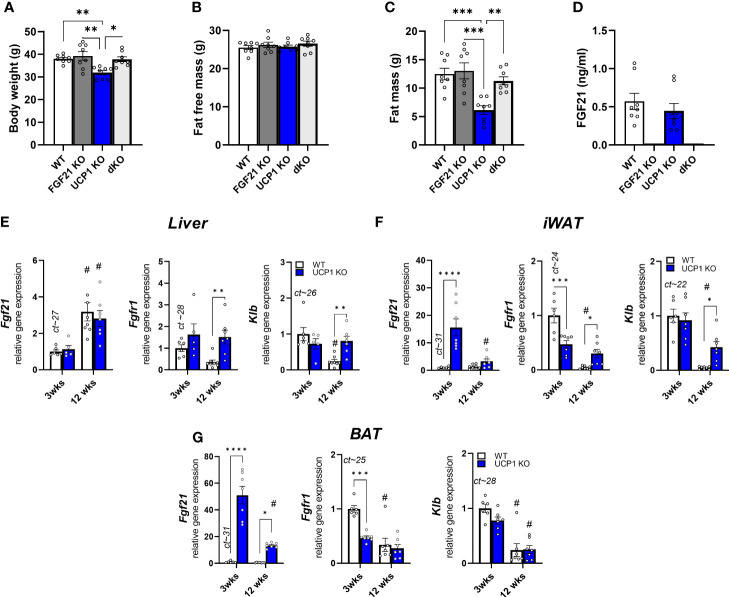
Sustained FGF21 receptor complex expression in UCP1 KO mice despite the lack of genotype difference in circulating FGF21 after long term HFD **(A)** Body weight, **(B)** fat free mass, **(C)** body fat, **(D)** serum FGF21 levels of WT, FGF21 KO, UCP1 KO and dKO mice fed a high fat diet for 12 wks. Relative gene expression of FGF21, Klb (beta klotho) and Fgfr1 (Fgf receptor 1) in **(E)** liver, **(F)** iWAT (inguinal white fat) and **(G)** BAT (brown fat) of WT and UCP1 KO mice fed a high fat diet for 3 or 12 weeks. Mean ct values of WT 3 weeks are given above the corresponding bar graph for quantitative assessment of gene expression. **(D)** Data are mean ± SEM.; n= 5-8. Statistical significance between genotypes are denoted by *p < 0.05, **p < 0.01, ***p < 0.001, ****p < 0.0001. Statistical differences between the treatment within one genotype are indicated #.

As shown previously (32), UCP1 KO mice are resistant to HFD feeding at room temperature, showing the same fat free mass, but reduced fat mass after 12 weeks of dietary treatment (**Figures 1A–C**). The obesity resistance is dependent on FGF21, as UCP1/FGF21 dKO mice gain similar body weight and fat mass as WT and FGF21 KO control mice (**Figures 1A, C**). Previously, we observed an early increase (after 3 weeks of HFD feeding) of circulating FGF21 in UCP1 KO mice, which was more than doubled compared to WT controls (32). In contrast, after long term HFD feeding, no differences between WT and UCP1 KO mice in serum FGF21 could be detected anymore **(Figure 1D**), as both genotypes show increased circulating FGF21 levels. On gene expression level, liver *Fgf21* is induced in WT and UCP1 KO mice in response to long term HFD (**Figure 1E**), independent of the differences in body weight (**Figure 1A**). However, as seen previously (32), in 3wks fed UCP1 KO mice *Fgf21* is additional induced in BAT and iWAT compared to WT (**Figures 1F, G**). Whereas the differences in iWAT disappear over time, the effect in BAT persists after 12 weeks HFD, but is less pronounced (**Figures 1F, G**). Despite having similar circulating FGF21 levels (**Figure 1D**), UCP1 KO mice still stay lean compared to WT and UCP1/FGF21 dKO mice, suggesting a higher, sustained FGF21 sensitivity. To get more insights, we next measured the gene expression of the FGF21 receptor complex in different tissues. In BAT, 12 weeks HFD led to a similar downregulation of *Klb* and *Fgfr1* in both genotypes compared to 3 weeks of HFD. In contrast, in liver and iWAT prolonged HFD feeding leads to a suppression of *Klb* and *Fgfr1* only in WT mice (**Figures 1E, F**), not in UCP1 KO mice, which still show sustained expression after 12 weeks HFD treatment (**Figures 1E, F**). Especially in iWAT, gene expression of FGF21 receptor complexes strongly downregulated in WT animals. Together, those data suggest a higher FGF21 sensitivity in liver and iWAT of UCP1 KO mice, which possibly could contribute to the lean phenotype.

### Higher sensitivity to exogenous FGF21 in UCP1 KO mice

**Figure 2:**
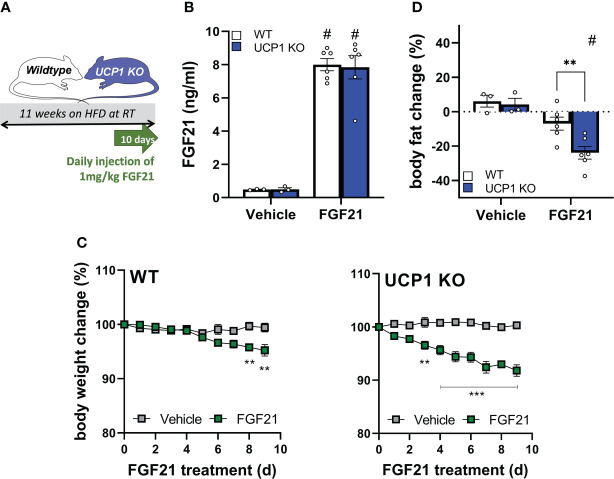
Higher sensitivity to exogenous FGF21 in UCP1 KO mice. **(A)** Scheme of the study design. **(B)** Serum FGF21 levels of WT and UCP1 KO mice 2-3 hours after injection with vehicle (0.9% NaCl) or 1mg/kg FGF21. **(C)** Body weight change over 9 days of WT and UCP1 KO mice treated once daily with NaCl or 1mg/kg FGF21. **(D)** Body fat change after 9 days of treatment. Data are mean+/-SEM; n= 3-6. Statistical significance between genotypes are denoted by **p < 0.01, ***p < 0.001. Statistical differences between the treatment within one genotype are indicated #.

With the discovery of similar circulating FGF21 levels after 12 weeks of HFD feeding at room temperature between UCP1 KO and WT mice (**Figure 1D**), we next tested if the DIO-resistant phenotype of UCP1 KO mice is due to higher FGF21 sensitivity. We performed a pharmacological study administrating 1mg/kg/day FGF21 for 10 days to long-term HFD fed WT and UCP1 KO mice (kept at room temperature; study design **Figure 2A**). As expected from **Figure 1D**, the saline treated control animals show the same FGF21 serum levels at the end of the treatment, whereas FGF21 injection leads to supra-physiological 16-fold induced circulating FGF21 levels in both genotypes (**Figure 2B**). Already after 3 days of FGF21 administration, UCP1 KO mice show significant weight loss (**Figure 2C**), whereas WT mice appeared to be resistant against FGF21 treatment in the initial phase of the treatment (**Figure 2C**), and only start losing significant body weight after 8 days of treatment. The stronger response to exogenous FGF21 in UCP1 KO mice is reflected in a significant body fat loss compared to WT mice at the end of the 10 days treatment period (**Figure 2D**). Together, these data support the hypothesis that UCP1 KO mice have a higher sensitivity to circulating endogenous and administrated FGF21 compared to WT.

### FGF21-induced weight loss coincides with FGF21 sensitivity in inguinal WAT but not in liver

**Figure 3:**
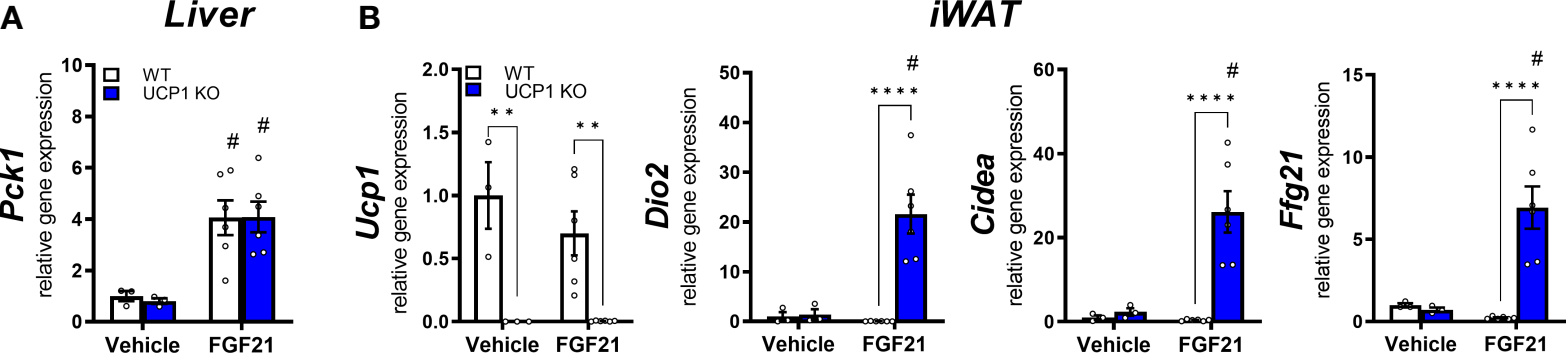
FGF21-induced weight loss coincides with FGF21 sensitivity in inguinal WAT but not in liver. Relative gene expression in **(A)** liver and **(B)** iWAT of WT and UCP1 KO mice fed a high fat diet for 10 weeks followed by a daily injection of vehicle (0.9% NaCl) or 1mg/kg FGF21 for 10 days. Data are mean ± SEM.; n= 3-6. Statistical significance between genotypes are denoted by **p < 0.01, ****p < 0.0001. Statistical differences between the treatment within one genotype are indicated #.

WT animals showed a reduced gene expression of the FGF21 receptor complex *Klb* and *Fgfr1* in liver and iWAT (**Figures 1E, F**). Furthermore, in the follow-up pharmacological study, WT animals showed a delayed response in body weight loss after exogenous administration of FGF21 compared to UCP1 KO mice (**Figure 2C**). Both datasets suggest the initiation of (tissue-specific) FGF21 resistance. Therefore, we next analysed gene expression of known FGF21 downstream targets in liver and iWAT of both genotypes after FGF21 administration. In liver, *Pck1* is upregulated in both genotypes (**Figure 3A**). However, in iWAT only UCP1 KO mice show an induction of *Dio2*, and *Cidea* after FGF21 injection (**Figure 3B**). Interestingly, FGF21 injection leads also to a strong upregulation of *Fgf21* gene expression itself in iWAT. These responses were completely blunted in WT mice (**Figure 3B**). Together, these data suggest iWAT as a key tissue regulating FGF21-mediated metabolic improvements in UCP1 KO mice and adipose tissue specific FGF21 resistance in WT mice.

## Discussion

Contrary to the metabolic improvements by FGF21 administration, endogenous FGF21 serum levels are increased during obesity-related diseases, suggesting FGF21 resistance. Whether obesity or adaptation to increasing metabolic stress provokes compensatory upregulation of FGF21, causing FGF21 resistance, is still an open question. Thus, the physiological role for obesity-induced FGF21 levels, if any, is not clear. Our data emphasize the importance of maintaining FGF21 sensitivity in adipose tissue during obesity to resist metabolic complications, as seen in UCP1 KO mice, which remain FGF21-sensitive in iWAT despite increased endogenous FGF21 levels. In contrast to WT mice, this sensitivity enables rapid weight loss and genetic responses in iWAT upon FGF21 administration.

Obesity is linked to leptin and insulin resistance, and even though the mechanism and response to pharmacological administration differs, it is not surprising that resistance to another circulating metabolic regulator, FGF21, has been suggested. Pioneering studies on FGF21 resistance in obese mice showed reduced receptor complexes and FGF21-signalling responses in WAT (19), suggesting an important role of adipose tissue mediating the metabolic effects of FGF21. Research groups have focused their efforts on increasing *Klb* expression to regain FGF21 responses, based on the essential role of KLB for FGF21 signaling (4, 5). Notably, adipocyte-specific deletion of *Fgfr1* or *Klb* eliminates the therapeutic benefits of FGF21 administration (30, 31). Unfortunately, controversial results of various adipose tissue specific KLB mouse models (25, 29) prevent unambiguous conclusions. A common observation in all studies, however, is the downregulation of the FGF21 receptor complex in adipose tissue during obesity (19, 25, 29, 35). Similar results were observed in a clinical study (28). Furthermore, in a seasonal model of obesity, the Siberian hamster, a reduction in adipose tissue *Klb* expression was associated with loss of ERK1/2 phosphorylation, altering responsiveness to FGF21 (36). Coherent with these previous observations, our study confirms repressed *Klb* and *Fgfr1* expression not only in iWAT, but also in BAT and liver of WT animals after prolonged high fat diet feeding. While after 3 weeks of HFD, *Klb* and *Fgfr1* are expressed in iWAT of WT mice, the mRNA levels strongly decrease after 12 weeks. In UCP1 KO mice, which display FGF21 dependent DIO resistance, the gene expression of the FGF21 receptor complex is sustained in the liver and in iWAT. By administration of exogenous FGF21 after 12 weeks of HFD, we showed that FGF21 signaling in the liver was still intact in both WT and UCP1 KO mice, while iWAT of WT mice does not respond to FGF21 injection. In our previous publication (32), we performed next generation RNA sequencing in several peripheral tissues to gain molecular insights on how FGF21 controls the obesity resistance in UCP1 KO mice. The strongest difference between WT and UCP1 KO mice was seen in iWAT, resembling a browning phenotype despite the absence of UCP1 (32). The present pharmacological study supports this finding by also pointing towards iWAT as the main target tissue of FGF21 action. In iWAT, FGF21 injection leads to a strong induction of FGF21-related gene expression only in UCP1 KO mice, suggesting a sustained FGF21 sensitivity in those mice. Furthermore, in iWAT of UCP1 KO mice FGF21 injection leads to a strong upregulation of *Fgf21* gene expression itself. Recently it was shown that browning of iWAT by β-adrenergic agonists requires autocrine FGF21 signaling to stimulation of thermogenic gene expression (37, 38), raising the question of the role of auto/paracrine versus endocrine signaling in the lean phenotype of UCP1 KO mice. Nevertheless, these findings indicate that obesity-induced downregulation of the receptor complexes can reduce tissue specific FGF21 responsiveness/sensitivity. Current research has emphasised to improve FGF21 signalling with exercise by subjecting mice to treadmill training for 3 days or 4 weeks during HFD feeding. Trained mice showed improved metabolic features compared to sedentary animals upon FGF21 injection (24). Using an adipose tissue-specific KLB knock-out model, it was shown that metabolism improved to a lesser extent, suggesting that the benefits of exercise are at least in part regulated by FGF21 in adipose tissues (24). Furthermore, bariatric surgery seems to improve FGF21 sensitivity during high-fat diets by restoring *Klb* expression in WAT and downregulating hepatic FGF21 expression in streptozotocin-induced diabetic rats (39), further supporting a key role for FGF21 signalling in WAT during metabolic disease.

In conclusion, our findings are coherent with recent research by supporting the concept of requiring sustained FGF21-sensitivity in adipose tissue for metabolic improvements during obesity and insulin resistance. However, the mechanisms of body weight loss are unclear. Previously it was shown that FGF21 administration to UCP1 KO mice can affect both, energy expenditure and food intake (40, 41). A detailed analysis of the energy balance during FGF21 treatment would be required to draw clear conclusions. Furthermore, the molecular mechanisms of increased iWAT FGF21 sensitivity improving body weight development and metabolic homeostasis are still unknown. In our previous work, we saw indications for upregulated (futile) lipid cycling in iWAT of UCP1 KO mice (32, 42), indicating a potential alternative thermogenic pathway, with a leaner phenotype as consequence. Furthermore, the induced browning phenotype in iWAT suggests increase mitochondrial content potentially impacting lipid metabolism. However, these phenomena need to be further elucidated in the future. Additionally, more research is needed to distinguish between endocrine versus para/autocrine effects of FGF21 signaling in iWAT. Keeping the pleiotropic role of FGF21 in mind, we cannot rule out that part of the effects could be mediated through central regulation *via* the brain. Thus, further investigations are required to understand and identify the pathways of dysregulated FGF21 signalling in obesity, which at the end will be important for the clinical translation of FGF21-based therapies.

## Data availability statement

The original contributions presented in the study are included in the article. Further inquiries can be directed to the corresponding author.

## Ethics statement

The animal study was reviewed and approved by local animal ethics committee of the state of Bavaria (Regierung Oberbayern) and North Stockholm.

## Author contributions

SK conceptualized the project, designed and performed experiments, interpreted data, and wrote the paper. MKH performed experiments and interpreted data, proof-read, commented and edited the manuscript. All authors contributed to the article and approved the submitted version.

## Funding

This work was supported by funding from the EFSD/Novo Nordisk Programme for Diabetes Research in Europe and Swedish Research Council (2018-02150).

## Acknowledgments

We would like to thank Daniel Brandt for excellent technical assistance and Martin Jastroch for helpful discussions. Furthermore, we would like to thank Brian Finan for providing the hFGF21.

## Conflict of interest

The authors declare that the research was conducted in the absence of any commercial or financial relationships that could be construed as a potential conflict of interest.

## Publisher’s note

All claims expressed in this article are solely those of the authors and do not necessarily represent those of their affiliated organizations, or those of the publisher, the editors and the reviewers. Any product that may be evaluated in this article, or claim that may be made by its manufacturer, is not guaranteed or endorsed by the publisher.
